# Transcriptomic profiling of sesame during waterlogging and recovery

**DOI:** 10.1038/s41597-019-0226-z

**Published:** 2019-10-15

**Authors:** Komivi Dossa, Jun You, Linhai Wang, Yanxin Zhang, Donghua Li, Rong Zhou, Jingyin Yu, Xin Wei, Xiaodong Zhu, Shiyang Jiang, Yuan Gao, Marie A. Mmadi, Xiurong Zhang

**Affiliations:** 1 Oil Crops Research Institute of the Chinese Academy of Agricultural Sciences, Key Laboratory of Biology and Genetic Improvement of Oil Crops, Ministry of Agriculture, No.2 Xudong 2nd Road, Wuhan, 430062 China; 2 College of Life Sciences, Shanghai Normal University, Shanghai, 200234 China

**Keywords:** Transcriptomics, High-throughput screening, Flooding, RNA sequencing

## Abstract

Sesame is naturally adapted to arid environments but highly susceptible to waterlogging stress. A few hours of waterlogging (lasting over 36 h) are detrimental to the crop growth, yield and survival. To better understand the molecular mechanisms underlying sesame responses to waterlogging and recovery, it is essential to design a high-resolution time-series experiment. In this study, we reported the RNA-seq profiling of two contrasting genotypes under waterlogging and recovery. The plants were grown in pots and subjected to waterlogging treatment at the flowering stage for 36 h and subsequently, 12 h drainage. Root samples were collected in triplicate at 22 time points under waterlogging/drainage treatments and at 10 time points in the control condition. This represents a total of 195 biological samples and the RNA-seq yielded over eight billion reads. Basic data analyses demonstrated a clear separation of transcriptomes from control, waterlogging and drainage treatments. Overall, the generated high-quality and comprehensive RNA-seq resources will undoubtedly advance our understanding of waterlogging/drainage responses in a non-model sensitive crop.

## Abstract (summary)

**Table:** 

Measurement(s)	transcription profiling assay • gene expression data
Technology Type(s)	RNA sequencing
Factor Type(s)	sampling time point • experimental condition
Sample Characteristic - Organism	Sesamum indicum

Machine-accessible metadata file describing the reported data: 10.6084/m9.figshare.9882866

## Background & Summary

With the recent surge in flooding events in many regions of the world, waterlogging has become a serious problem for agricultural production^1^. Soil waterlogging leads to hypoxia, high CO_2_ in the root zone and slows down photosynthesis, which ultimately impairs normal crop growth and yields. Like most of typical dryland crops, sesame (*Sesamum indicum* L.) is highly sensitive to waterlogging stress^2^. Waterlogging occurring in several sesame growing areas in Asia and Africa engenders catastrophic economic loss for smallholders. According to Sun *et al*.^3^, most of the sesame cultivars hardly survive over 36 h of waterlogging in the fields. Previous studies that analyzed sesame transcriptional responses to waterlogging and drainage were conducted with limited temporal resolution^4,5^, hence, the architecture and dynamics of the waterlogging/recovery gene regulatory network are yet to be elucidated. The present study generated high-quality and high-resolution time series RNA sequencing data (195 RNA-seq in total) from root of two contrasting sesame cultivars (ZZM2541 and Ezhi-2) during the waterlogging and recovery stages. We believe that these precious resources from a non-model crop will help unlock novel genes-pathways-mechanisms modulating waterlogging/drainage responses and assist in crop improvement strategies.

## Methods

### Plant materials and stress treatment

**Fig. 1:**
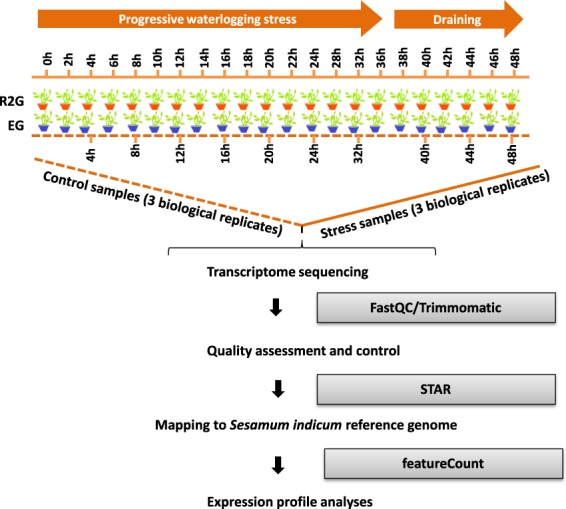
Flowchart of experimental design of this study. A high-resolution temporal waterlogging/drainage treatment was used to capture the gene expression changes in roots of two *S*. *indicum* genotypes (EG and R2G). The untreated plants were used as controls. Three biological replicates per condition were sampled for transcriptome sequencing. All raw reads were quality controlled prior to aligning to *S*. *indicum* reference genome (v1.0). The uniquely aligned reads were employed for expression profile analyses.

Two genotypes of sesame (*Sesamum indicum* L.) were obtained from the China National Genebank, Oil Crops Research Institute, Chinese Academy of Agricultural Sciences and used in this experiment. The genotype ZZM2541 (R2G) displays a strong tolerance to waterlogging stress while Ezhi No. 2 (EG) is highly susceptible as demonstrated by Wei *et al*.^2^. The experiment was conducted in a greenhouse as described by Wang *et al*.^5^ and Dossa *et al*.^6^. Plants were grown in pots (25 cm diameter and 30 cm depth) containing 7 Kg of loam soil mixed with 10% compound fertilizer. The plants were irrigated every 3 days and the soil volumetric water content (vwc) was maintained at ~35%. A pot tray was placed under each pot to avoid water loss. A completely randomized blocking design with 3 replicates was employed. 15 days after the initiation of flowering, half of the pots were waterlogged by standing in a plastic bucket filled with tap water up to 3 cm above the soil surface. Each pot contains 3 seedlings, which were maintained waterlogged for 36 h and afterwards, pots were drained to allow plants to recover for 12 h (vwc = ~35%). In parallel, half of the pots were kept under normal growth conditions (vwc = ~35%) during the whole experiment. Root samples were collected from a single plant/pot from the three replicated pots (3 biological replicates) in the stress and control treatments at the different time points following the flowchart presented in Fig. 1. After 48 h treatment, several EG plants were dead while few survived. We therefore sampled separately both dead (EG-48-W) and survived (EG-48) EG plants. In total, 195 root samples were collected and snap frozen in liquid nitrogen for follow-up analyses (Sample information for the study) available at figshare^7^.

### RNA extraction, library preparation and RNA sequencing

Total RNA was extracted from 195 root samples using the TRIzol reagent (Invitrogen), and treated with DNase I and Oligo (dT) to isolate mRNAs. The concentration and quality were determined using an ultraviolet spectrophotometer and 2% denaturing agarose gels. The cDNA was synthesized using the mRNA fragments as templates. The short fragments (~300 bp) were ligated with adapters and the suitable fragments were selected for PCR amplification. The libraries were paired-end sequenced using the Illumina platform Hiseq 2500^8^.

### RNA-seq data processing

**Fig. 2:**
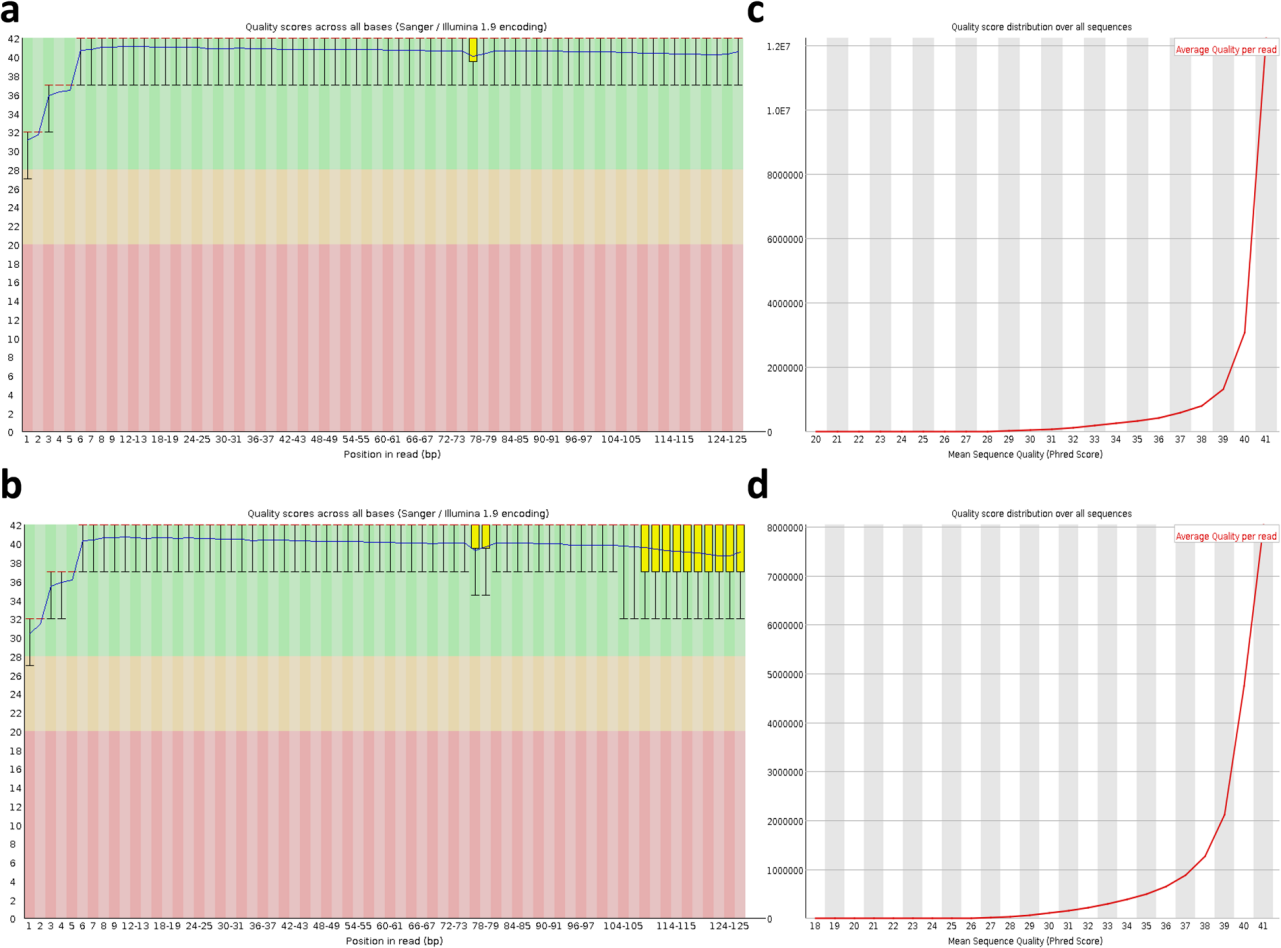
RNA sequencing quality details. An example of the FastQC report illustrating the average quality scores across all bases of the paired-end datasets for the sample CK_EG_12_rep1 (SRR8490161). (**a**,**b**) Phred quality scores for each nucleotide position are represented as a box and whisker plot. The central red line is the median value. The yellow box represents the inter-quartile range (25–75%). The upper and lower whiskers represent the 10 and 90% points. The blue line represents the mean quality. (**c**,**d**) Average quality per read along the reads.

The program FastQC (http://www.bioinformatics.babraham.ac.uk/projects/fastqc/) was employed to determine the base quality of the raw reads (in FASTQ format) and we removed the paired-end reads containing more than 5% ambiguous residues (Ns) and those containing >10% bases with a Phred quality score of 10 (Fig. 2). Then, the raw reads were trimmed using Trimmomatic, version 0.32^9^. After cleaning and quality reassessment with FastQC, approximately 31.6–56.8 million high-quality reads of 90-bp length remained in each sample (Quality check report of 195 sesame transcriptomes under control, waterlogging and drainage treatments) available at figshare^7^. The high-quality reads were mapped to the sesame (*Sesamum indicum* L.) reference genome v1.0 (http://ocri-genomics.org/Sinbase/login.htm)^10^ using the STAR software^11^, allowing no more than one mis-match in the alignment. Approximately, 88.9–97.4% of the clean reads were uniquely mapped to the reference genome, with 94.3–98.6% of them uniquely mapped to the genic regions (Statistics of the clean read mapping) available at figshare^7^. Using the featureCount package^12^, the gene expression levels were calculated based on the number of unique matched reads to the sesame genome v1.0^10^ and were normalized to Transcripts Per Million (TPM).

## Data Records

The RNA-seq raw data of the 195 samples are deposited to the National Center for Biotechnology Information (NCBI) Sequence Read Archive (SRA) under the accession SRP181800^13^. The gene expression data of the 195 RNA-seq samples are deposited to the Gene Expression Omnibus under the accession GSE133186^14^. Supplementary files accompanying this manuscript have been deposited to figshare^7^.

## Technical Validation

### Quality control

In this project, a total of 195 RNA libraries were prepared and sequenced generating over 8 billion raw reads (Quality check report of 195 sesame transcriptomes under control, waterlogging and drainage treatments) available at figshare^7^. By applying FastQC to the whole dataset, we successfully obtained high quality clean data with 97% of bases scoring Q30 and above (Fig. 2). Approximately, 98% of reads could be uniquely mapped to the reference genome of *S*. *indicum*.

### Basic analysis of RNA-seq data

**Fig. 3:**
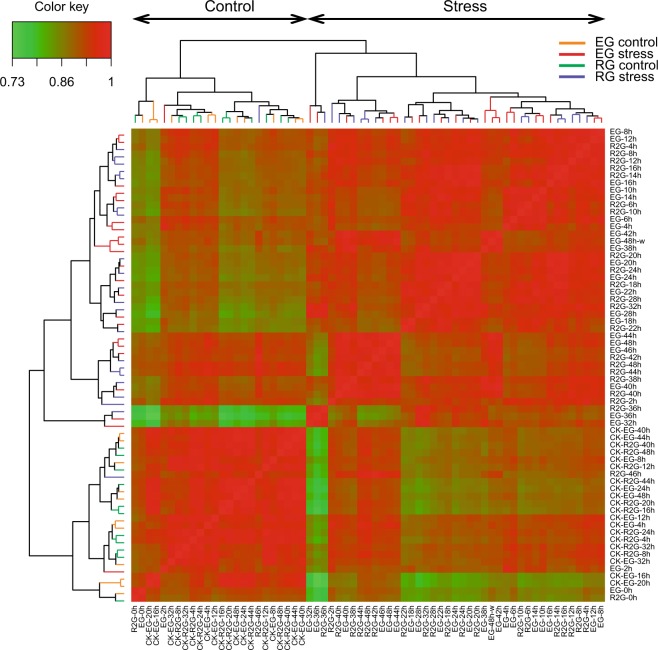
Heatmap clustering of the transcriptome data. Correlations were estimated using the Pearson correlation coefficient based on TPM values. In total two main groups of samples were obtained, including control and stressed samples.

**Fig. 4:**
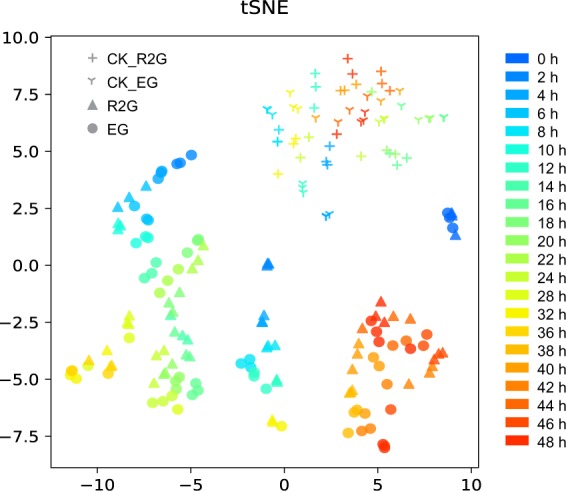
Tsne scatter plot of the samples. The plot depicts the clustering patterns of the samples according to the genotypes and stress treatments. In the diagram, the transverse ordinates represent the first and second principal components; the symbols in the graph represent the samples, and the different colors represent the time points of sampling.

A heatmap for cluster relationships among the samples representing Pearson distance was generated with the TPM values using the R package ‘*Pheatmap*’ v.1.0.12 (https://cran.r-project.org/web/packages/pheatmap/index.html) (Fig. 3). It could be observed that samples from the control conditions (CK) and those from the stress treatments formed distinct groups. To further assess sample relationships and the time-course expression patterns, we implemented the tsne reduction scatter plot, which is a non-linear dimensionality reduction method for embedding high dimensional data into a low-dimensional space^15^. The analysis was performed with the R package ‘*tsne’* v.0.1–3. The results showed a clear separation between samples from control (CK), waterlogging treatment (0-36 h) and drainage phase (36–48 h) (Fig. 4). In addition, the difference between the two contrasting genotypes used in this study could be observed through their differential gene expression during the waterlogging treatment.

## Usage Notes

This study generated dense and high-resolution gene expression data under waterlogging stress-drainage in a non-model crop. In contrast to many experimental designs relating to temporal transcriptome profiling under stress which set the beginning of the stress application as the control, in the present project, we collected samples at different time points under the control condition. Comparing expression data from samples harvested at the same time point under stress and control conditions will provide more accurate differential gene expression records. Concerning the time points for which samples were uniquely harvested under stress treatments, the users can still use the previous time point in the control condition for gene differential expression analysis. The whole datasets were publicly deposited at NCBI SRA and we anticipate that our RNA-seq data would provide new insights into the molecular basis of waterlogging stress responses in plants.

## Data Availability

Codes that were used for the RNA-seq data processing are available at figshare^7^. Software and their versions were described in Methods.
